# The evolving role of platelets in aging and cancer progression

**DOI:** 10.3389/fcell.2026.1946388

**Published:** 2026-10-30

**Authors:** Si-Yu Huang, Ming-Shun Zhang, Ze-Sen Feng, Wei Cui, Fu-Hui Xiao

**Affiliations:** Department of Colorectal Surgery, The Affiliated Lihuili Hospital of Ningbo University, School of Medicine, Ningbo University, Ningbo, Zhejiang, China

**Keywords:** aging, cancer, hyperreactivity, platelet, senescence

## Abstract

Platelets, once considered solely for their roles in hemostasis and thrombosis, are now recognized as pivotal players in the aging process and cancer progression. Aging reprograms the platelet compartment at multiple levels, including reduced counts, structural alterations, shifts in transcriptomic and proteomic profiles, fostering a hyperactive and pro-inflammatory state termed thromboinflammation. Platelets not only contribute to age-related cardiovascular and inflammatory disorders but also are involved in tumorigenesis. In the circulation, platelets bind to circulating tumor cells and form a physical barrier that protects them from immune destruction. Within the tumor microenvironment, platelets subsequently facilitate cancer progression by orchestrating angiogenesis, enhancing metastatic dissemination, and suppressing anti-tumor immunity. Platelets also give rise to tumor-educated platelets (TEPs) that carry diagnostic molecular signatures. This review systematically delineates the dynamic functions of platelets in aging and in cancer, with a brief discussion on the potential link between aged platelets and cancer progression. We also discuss the potential value and future directions of platelet-targeted therapeutic strategies in the prevention and treatment of age-related cancers.

## 1 Introduction

Platelets are the smallest anucleate cellular components in the circulation, appearing as disc-shaped fragments with a diameter of approximately 2–5 μm (Thon and Italiano, 2012; Tian et al., 2025). The platelet life cycle is displayed in Figure 1. In brief, they are primarily and continuously produced by megakaryocytes (MKs) in the bone marrow through a sophisticated process known as thrombopoiesis (Carminita et al., 2024), which is initiated by hematopoietic stem cell differentiation and tightly regulated by thrombopoietin (TPO) (Machlus and Italiano, 2013). During maturation, MKs undergo endomitosis, characterized by DNA replication without cytokinesis, resulting in polyploidization and cytoplasmic expansion, accompanied by the formation of an invaginated membrane system (IMS) that supplies membrane components for platelet formation and the biogenesis of platelet-specific organelles. In the final state of thrombopoiesis, MKs extend cytoskeleton-driven proplatelet processes into the sinusoidal vasculature, where shear forces within the circulation subsequently mediate fragmentation of these extensions, giving rise to mature circulating platelets. Platelets have a relatively short lifespan, circulating in human blood for 7–10 days. During aging, platelets undergo progressive structural and metabolic changes that lead to the exposure of clearance signals, including desialylation and phosphatidylserine (PS) externalization (Dasgupta et al., 2010; Grozovsky et al., 2026). These signals promote their recognition and removal primarily by the spleen and liver, ensuring efficient platelet turnover and homeostasis (Reusswig et al., 2024).

**FIGURE 1:**
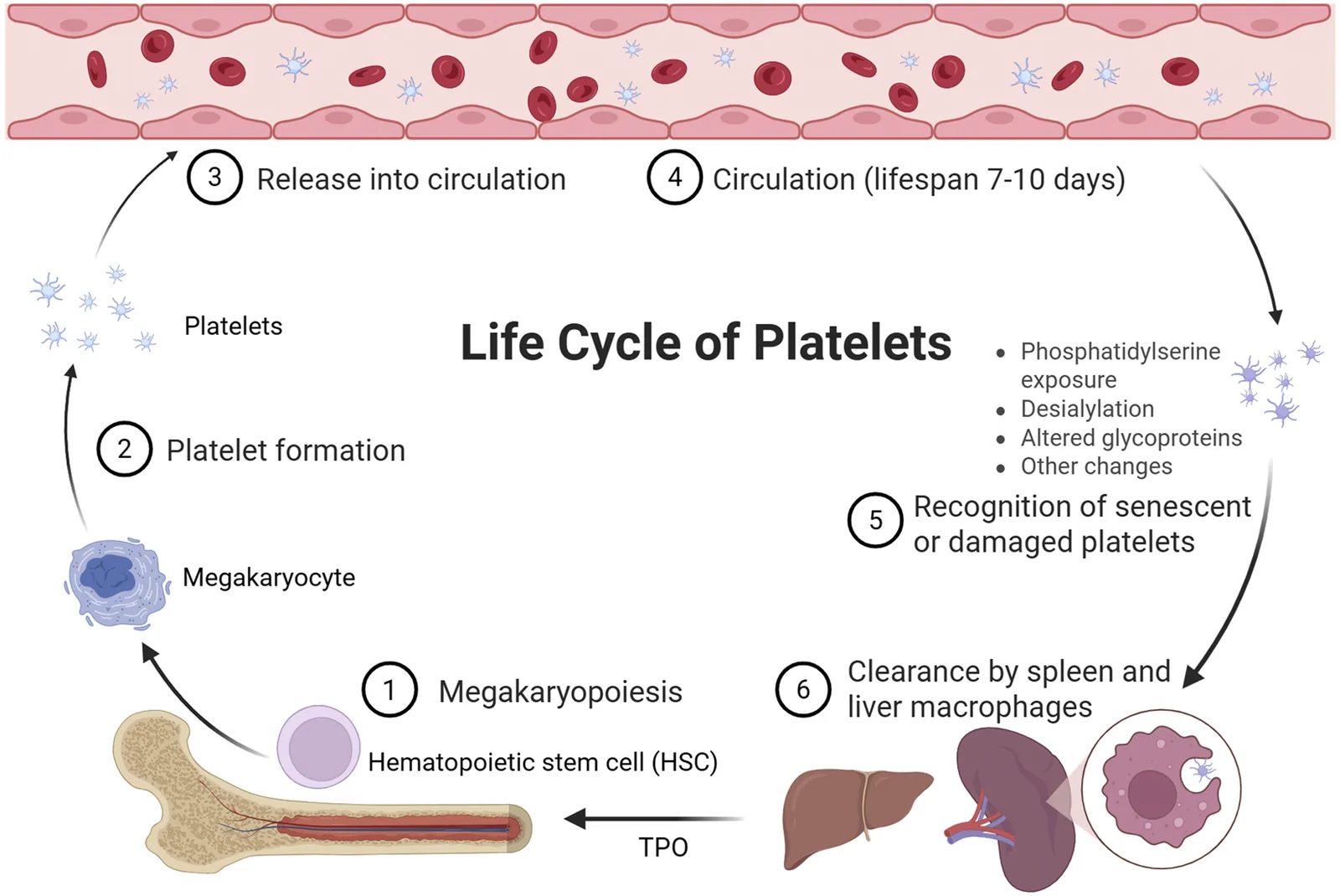
The life cycle of platelets: from production to clearance. This schematic illustrates the sequential stages of platelet life. In the bone marrow, hematopoietic stem cells (HSCs) differentiate into megakaryocytes under the regulation of hematopoietic growth factors, including TPO, which is secreted by the liver (1). Megakaryocytes extend proplatelets into bone marrow sinusoids, where platelets are released via cytoplasmic fragmentation (2). Newly formed platelets then enter the bloodstream (3) and circulate for approximately 7–10 days, surveilling vascular integrity and participating in hemostasis and thrombosis (4). As platelets age or become damaged, they expose “eat-me” signals (e.g., phosphatidylserine) and other alterations, which are recognized by clearance receptors (5). Finally, senescent or damaged platelets are cleared by macrophages in the spleen and liver (6). (Created with BioRender.com).

Platelets are highly specialized cytoplasmic fragments enriched with diverse bioactive components that support a broad range of functional outputs. They contain various organelles, including mitochondria, lysosomes, peroxisomes, and secretory organelles: α-granules and dense granules (Liu and Welburn, 2024; Yao and Kahr, 2025). Despite lacking a nucleus, platelets retain a functional RNA repertoire derived from megakaryocytes, including mRNAs and non-coding RNAs, which possess functions such as splicing and translation, and enable adaptive responses upon activation (Mills et al., 2017). In addition, proteomic analyses have revealed that platelets contain thousands of proteins, including coagulation factors, cytokines, chemokines, growth factors, and immunomodulatory molecules, forming the material basis for their multifunctionality (Burkhart et al., 2014; Huang et al., 2021; Yao and Kahr, 2025). Notably, platelet molecular composition is dynamically influenced by systemic conditions, including aging, cancer, and other physiological variables (Best et al., 2015; Angénieux et al., 2016; Supernat et al., 2021), highlighting their high degree of functional plasticity.

Platelets have long been recognized as key mediators of hemostasis, owing to their ability to adhere to the damaged vascular wall and form aggregates (Brewer, 2006). Upon vascular endothelial injury, they adhere to exposed collagen through surface receptors such as the glycoprotein (GP) Ib-IX-V complex. After activation, platelets undergo morphological changes and release mediators such as adenosine diphosphate (ADP) and thromboxane A2 (TXA2), promoting aggregation and thrombus formation (Koupenova et al., 2018; Scridon, 2022). Beyond hemostasis, platelets actively participate in immune and inflammatory processes. For instance, they express immune-related molecules such as Toll-like receptors (TLRs) and Fc receptors, and secrete mediators such as IL-1β and CD40L, thereby directly interacting with immune cells and regulating both innate and adaptive immune responses (Semple et al., 2011; Koupenova et al., 2022; Karakas and Ni, 2024). Under infectious or inflammatory conditions, platelets can recognize pathogens, enhance leukocyte recruitment, and influence the intensity of cytokine storms, thus playing a dual role in immune defense and inflammatory pathology (Hottz et al., 2020). Consequently, platelets are now recognized as multifunctional regulators involved in inflammation, host defense and tissue repair (Li et al., 2024; Bo and Zhao, 2025; Hu et al., 2025; Kusch et al., 2026).

Importantly, accumulating evidence supports that platelet biology is not static but dynamically remodeled across the lifespan. Aging is associated with systemic inflammatory and metabolic alterations that reshape platelet reactivity and molecular composition, whereas in cancer, platelets are actively reprogrammed by tumor-derived signals to promote tumor growth, immune evasion, and metastasis. These observations position platelets as central nodes linking hemostasis, aging, immunity, and cancer progression.

## 2 Aging-associated remodeling of platelet phenotype and function

The aging of the platelet compartment is a complex, multi-layered process involving both the chronological aging of the host (organismal aging) and the circulatory maturation of individual platelets (cellular senescence) (Figure 2). This remodeling encompasses significant shifts in systemic counts, structural integrity, and the molecular landscape, collectively dictating the transition from balanced hemostasis to age-related vascular pathologies. In the literature, the term “aged platelets” must be interpreted with caution, as it can refer to two distinct biological contexts. It may denote platelets obtained from older individuals (organismal aging), or alternatively, platelets at a late stage of their circulating lifespan. In this review, “aged platelets” refers primarily to platelets obtained from elderly individuals (organismal aging), whereas “senescent platelets” refers to late-stage platelets that exhibit senescence-associated features, including loss of sialylation and increased exposure of PS.

**FIGURE 2:**
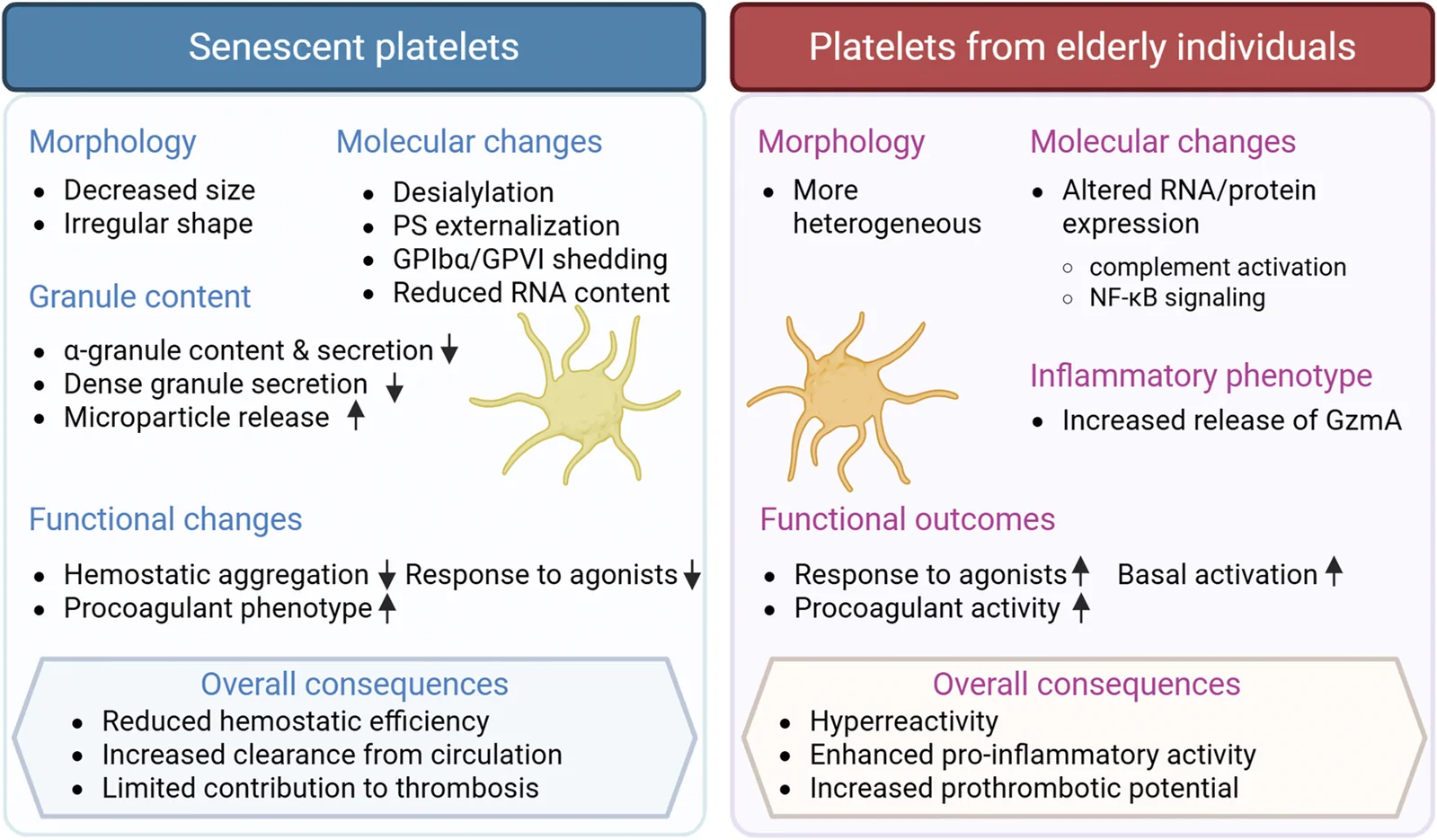
Characteristics of senescent platelets and age-associated changes in platelets from elderly individuals. Senescent platelets represent the intrinsic aging process occurring during the platelet lifespan, accompanied by progressive changes in morphology, molecular composition, and functional properties, ultimately proceeding toward platelet clearance. In contrast, platelets from elderly individuals exhibit age-associated alterations shaped by the systemic aging environment, including molecular changes and inflammatory status, which influence platelet activation, reactivity, and thrombotic potential. (Created with BioRender.com).

### 2.1 Changes in platelet count and structure

Studies from multiple ethnic human populations across the world have shown that the platelet count decreases in old age (Segal and Moliterno, 2006; Biino et al., 2013; Balduini and Noris, 2014). It is worth noting that this decrease does not follow a simple linear pattern across the lifespan. Platelet counts drop during childhood, stay relatively steady through middle age, and then fall again in the elderly (Segal and Moliterno, 2006; Jain et al., 2019; Biino et al., 2013). Moreover, there is a clear sex difference in platelet counts after childhood, with women consistently showing higher values than men, and this disparity continues into old age, where platelet counts are reduced by 35% in men and by 25% in women relative to early infancy (Biino et al., 2013). Although the decline in platelet counts in aged individuals is largely confirmed, the underlying mechanisms remain uncertain and are currently only a matter of theoretical interpretation, such as a reduction in hematopoietic stem cell reserve during aging or a survival advantage in individuals with lower platelet counts (Biino et al., 2013).

In addition to changes in platelet count, some population-based studies have reported a modest increase in mean platelet volume (MPV) in older individuals, which may reflect age-related alterations in platelet production and clearance dynamics (Lippi et al., 2012). However, studies examining platelet size at the single-cell level have reported that senescent platelets exhibit reduced volume, which is approximately half the cross-sectional area of young platelets (Hille et al., 2020). This apparent discrepancy likely reflects that MPV measures the entire circulating pool, which may be skewed toward younger platelets in elderly individuals, whereas single-cell analyses directly compare platelets of different ages within the same individual. Accumulating evidence suggests that platelet size and age are not strictly coupled, although both parameters can independently influence platelet function (Thompson et al., 1984). At the structural level, senescent platelets show reduced cytoskeletal density and complexity, as well as diminished mitochondrial content (Allan et al., 2021). In the elderly, studies have observed that platelet membranes become increasingly irregular and non-smooth, with multiple ruptures, while pseudopodia and protuberances grow more numerous and slender (Tian et al., 2019). Moreover, senescent platelets are associated with changes in cellular structure that may alter buoyant density (Schlesinger, 2018; Corash et al., 1984), which has been proposed as an indirect marker of platelet age (Corash et al., 1978; Mezzano et al., 1981).

### 2.2 Changes in platelet function

Senescent platelets, throughout their life cycle, typically display reduced RNA content and decreased reactivity (Pirabe et al., 2023). They are marked by shedding of surface receptors, including GPIbα and GPVI, which serve as a signal for their clearance from circulation (Qiao et al., 2010). Veninga et al. observed the association between GPVI expression and platelet aging, with elevated GPVI expression correlated with high RNA content and increased platelet reactivity (Veninga et al., 2022). Furthermore, senescent platelets also exhibit several functional changes, such as reduced α-granule content and secretion, diminished dense granule secretion, and a tendency toward increased microparticle release (Pienimaeki-Roemer et al., 2015; Allan et al., 2021). One study has revealed that senescent platelets lose their hemostatic proficiency but are more prone to react to inflammatory challenges, exhibiting increased formation of platelet-leukocyte aggregates, enhanced adhesion to neutrophils, and improved bactericidal function compared with reticulated platelets (Anjum et al., 2025). Consequently, they display decreased hemostatic aggregation and reduced responsiveness to agonists, whereas their procoagulant phenotype is enhanced (Montenont et al., 2019; Anjum et al., 2025).

At the organismal level, aging is a well-established independent risk factor for thrombosis and other cardiovascular diseases. This increased risk is largely attributed to a shift toward a hyperreactive platelet phenotype, the most notable age-associated functional change, characterized by enhanced platelet activation and reduced activation thresholds against agonists (Le Blanc and Lordkipanidzé, 2019). Consistent with this, Gnanenthiran et al. identified an aging-associated platelet phenotype including increased basal activation, enhanced responsiveness to ADP, and thrombin resistance (Gnanenthiran et al., 2022). At the molecular level, platelets from elderly individuals exhibit extensive transcriptomic and proteomic alterations involving pathways related to platelet degranulation, complement activation, and nuclear factor-κB (NF-κB) signaling pathway (Montenont et al., 2019; Chen et al., 2024). Aged individuals harbor platelets that release granzyme A (GzmA), which plays an important role in promoting inflammation, possibly through the induction of IL-8 (Campbell et al., 2018). In addition, emerging evidence indicates that platelets from elderly individuals may be more heterogeneous (Kaiser et al., 2025). The age-related heterogeneity may not only reflect gradual functional remodeling of existing platelets, but also involve alterations in the platelet production program. For instance, in parallel with canonical platelet production, an age-related ‘shortcut’ of the platelet differentiation cascade has been identified, which causes an additional, functionally hyperreactive platelet population (Poscablo et al., 2024). More recently, Jia et al. revealed that aging platelets exhibit a systemic reduction in global ubiquitination alongside the upregulation of ubiquitin-specific peptidase 25 (USP25), which drives elderly platelet hyperreactivity by deubiquitinating and stabilizing talin-1 (Jia et al., 2026).

### 2.3 Alterations in omic profiles

Healthy human platelets contain approximately 9,500 known protein-coding gene transcripts and over 4,000 types of proteins, and these molecular repertoires are dynamically changed in response to different stimuli (Burkhart et al., 2012; Bray et al., 2013; Gutmann et al., 2020). During aging, corresponding changes also occur in both the transcriptomic and proteomic profiles of platelets. At the RNA level, platelet RNA content decreases with age, and significant transcriptomic differences exist between young and senescent platelets (Angénieux et al., 2016; Allan et al., 2023). For instance, previous studies have shown that compared with reticulated (young) platelets, senescent platelets exhibit thousands of differentially expressed transcripts (Bongiovanni et al., 2019; Hille et al., 2020). Among these, genes involved in platelet activation and aggregation (e.g., ITGA2B, ITGB3, and SELP) are predominantly downregulated in senescent platelets, which may account for the reduced reactivity observed as platelets age (Hille et al., 2020; Bongiovanni et al., 2019). At the protein level, multiple studies have shown that anucleate platelets are capable of protein synthesis (Weyrich et al., 2009; Rowley and Weyrich, 2017). Under certain physiological stimuli, platelets can initiate translation and cause changes in the protein profile (Wurtzel et al., 2024). Some studies have found that the total protein content of senescent platelets significantly decreases compared to young platelets (Allan et al., 2021).

## 3 The complex roles of platelets in tumors

A growing number of studies have shown the roles of platelets in tumor biology. A bidirectional communication exists between platelets and tumor cells. Platelets facilitate tumor progression and metastasis through diverse mechanisms, such as modulation of proliferation, angiogenesis, epithelial-mesenchymal transition (EMT), immune evasion, and extravasation. Moreover, cancer significantly alters platelets in their count and composition, including mRNA, non-coding RNA, proteins, and lipids, leading to the formation of tumor-educated platelets (TEPs). Owing to their tumor-related molecular signatures, TEPs have emerged as promising biomarkers for liquid biopsies and cancer diagnosis.

In contrast to their well-documented pro-tumorigenic roles, accumulating evidence indicates that platelets can also exert anti-tumor functions. For instance, platelets have been shown to deliver pro-apoptotic molecules, such as miR-24, into solid tumor cells, thereby suppressing tumor growth (Michael et al., 2017). Additionally, platelets release immunomodulatory factors, including CD40L, which activates CD8^+^ T cell-mediated immune surveillance to inhibit tumor progression (Ma et al., 2022). In this review, we focus on the emerging potential of platelets as mediators of tumor progression (Figure 3).

**FIGURE 3:**
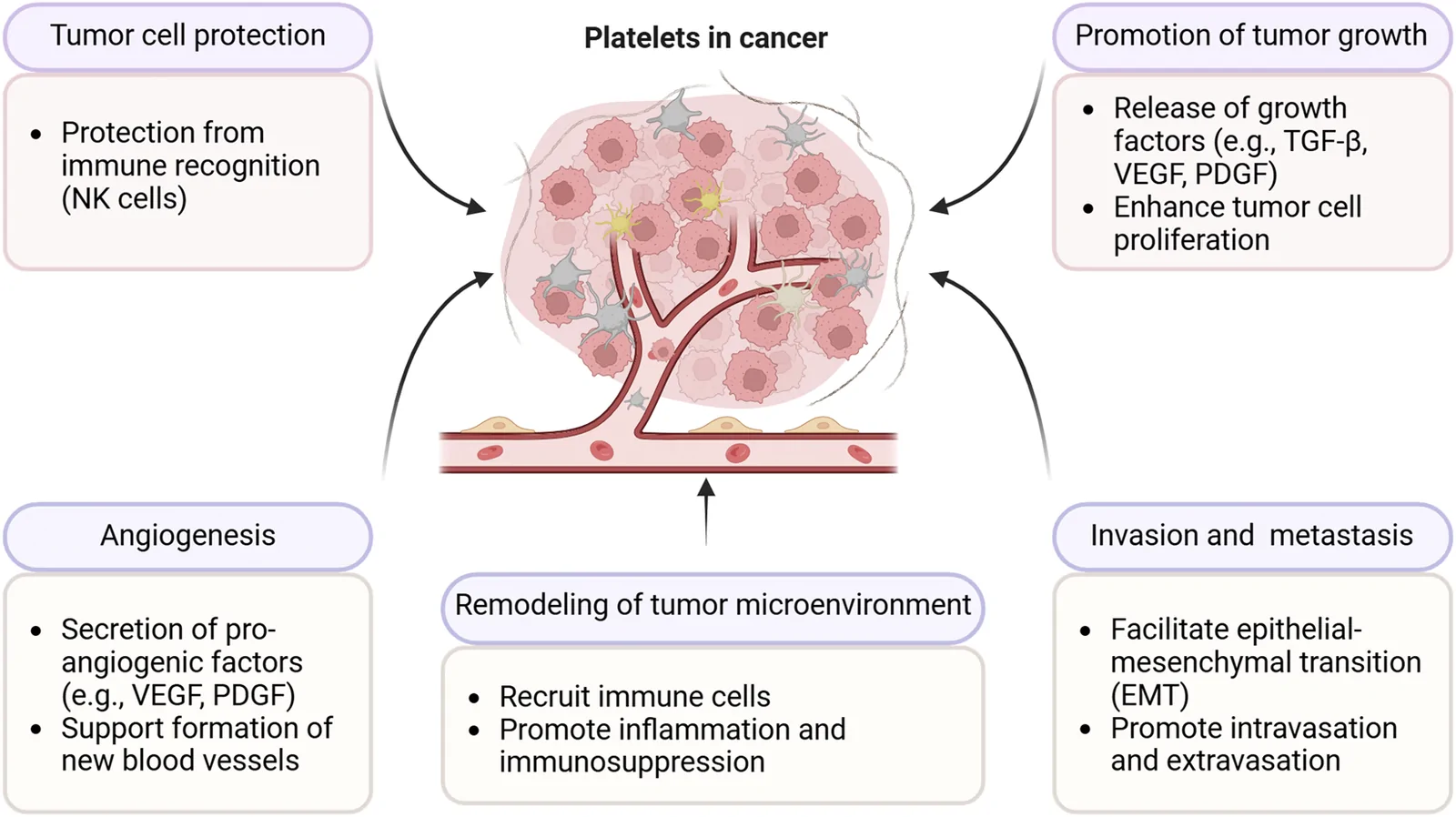
Platelet-mediated regulation of tumor progression. Platelets regulate tumor development through multifaceted interactions with cancer cells and the tumor microenvironment. Upon activation by tumor-associated signals, platelets release various bioactive molecules, such as growth factors and cytokines, which promote tumor cell proliferation, angiogenesis, invasion, and EMT. Platelets also facilitate metastatic dissemination by protecting circulating tumor cells, enhancing immune evasion, supporting tumor cell extravasation, etc. (Created with BioRender.com).

### 3.1 Platelets contribute to tumor growth and promote angiogenesis

Platelets are increasingly recognized as key circulating regulators of the tumor microenvironment, where they sustain the survival and development of primary tumor lesions through mechanisms such as the release of growth factors and the regulation of angiogenesis (Li et al., 2024). Upon activation, platelets undergo degranulation and release a diverse array of bioactive molecules that directly drive tumor cell proliferation. For example, previous studies have shown that tumor-derived factors, such as thrombin and ADP, can activate platelets through specific receptors (e.g., PARs and P2Y12) on the platelet surface, thereby triggering α-granule degranulation and the subsequent secretion of factors, such as TGF-β and IGF-1, which can directly activate the proliferation pathway of cancer cells (Rab et al., 2025).

Beyond directly regulating tumor cell growth, platelets are potent drivers of angiogenesis, the process by which new blood vessels form to supply oxygen and nutrients to developing tumors. Upon activation, platelets release numerous pro-angiogenic factors, including vascular endothelial growth factor (VEGF) and platelet-derived growth factor (PDGF), which promote endothelial cell sprouting, matrix activation and vascular remodeling. For instance, during vascular remodeling, PDGF released by platelets activates corresponding receptor PDGFR on interstitial cells and recruits pericytes and fibroblasts to cover new blood vessels (Raica and Cimpean, 2010). In addition, platelets also release matrix metalloproteinases (MMPs) that degrade the extracellular matrix, thereby facilitating the extension of new blood vessels (Gay and Felding-Habermann, 2011). Although platelets also harbor endogenous angiogenesis inhibitors, such as PF4, thrombospondin-1 and endostatin (Wojtukiewicz et al., 2017), some studies have suggested that tumor-derived signals may induce specific sorting mechanisms, leading to the preferential release of pro-angiogenic factors (Italiano et al., 2008). Studies have shown that platelet-derived extracellular vesicles (P-EVs) can be internalized by tumor cells, upregulating ITGB3 and activating the MAPK/ERK/ATF4/Nrf2 axis, thereby enhancing SLC7A11 expression and suppressing ferroptosis (Li et al., 2022).

### 3.2 Platelets are involved in epithelial-mesenchymal transition and tumor metastasis

A growing number of studies have shown that platelets play a crucial role in cancer metastasis through multiple mechanisms, including the induction of EMT, facilitation of circulating tumor cell (CTC) survival, and promotion of vascular extravasation. Tumor cells with high metastatic potential interact extensively with platelets in the bloodstream. Platelets can aggregate around tumor cells through adhesion molecules such as P-selectin and the GPIb-IX-V complex, which binds von Willebrand factor (vWF), as well as integrins that engage corresponding ligands on tumor cells (Schlesinger, 2018). This platelet-tumor cell interaction not only protects tumor cells from immune clearance but also amplifies TGF-β signaling, a central driver of EMT (Liu et al., 2021; Zhou et al., 2023). TGF-β1 activates the classic Smad pathway in tumor cells, downregulating epithelial markers such as E-cadherin while upregulating mesenchymal markers such as vimentin and N-cadherin. In parallel, platelet-mediated TGF-β1 can also trigger the activation of nuclear factor κB (NF-κB) pathway and facilitate the initiation of EMT (Labelle et al., 2011). In order to overcome the growth inhibitory effect of TGF-β in early tumors, platelets promote the transformation of tumor subclones to invasive phenotype through transient physical contact (Labelle et al., 2011).

Beyond EMT induction, platelets can facilitate CTC intravasation, extravasation, and survival in target organs. Integrin αIIbβ3 on platelets interacts with endothelial intercellular adhesion molecule-1 (ICAM-1) via a fibrinogen bridge, while the GPIb-IX-V complex binds vWF, collectively promoting firm adhesion of CTC to vascular endothelium (Bombeli et al., 1998). Platelet-derived ADP further disrupts the integrity of endothelial intercellular junctions through activation of the P2Y2 receptor on vascular endothelial cells, creating a permissive microenvironment for tumor cell extravasation (Schumacher et al., 2013). After extravasation, disseminated tumor cells encounter oxidative and metabolic stresses in the new tissue microenvironment, making them susceptible to ferroptosis, an iron-dependent form of cell death that acts as a barrier to metastasis (Ubellacker et al., 2020). In addition, activated platelets recruit neutrophils and induce them to release extracellular traps (NETs) through TLR4-ERK5 and other pathways, which also promote tumor metastasis (Ren et al., 2021).

### 3.3 Platelets as modulators of the tumor immune microenvironment

Platelets play a multifaceted role in shaping the tumor immune microenvironment, promoting immune evasion and supporting tumor progression. They interact with various immune cell populations and modulate their functions (Tuerhong et al., 2024), and also actively recruit immune cells, such as neutrophils (Li N. et al., 2026). Platelets are reservoirs of immunomodulatory molecules, including TGF-β and serotonin (5-hydroxytryptamine, 5-HT). Platelet-derived TGF-β, activated via glycoprotein A repetitions predominant (GARP), suppresses cytotoxic T cell function and fosters regulatory T cell expansion, thus promoting an immunosuppressive TME (Rachidi et al., 2017). Activated platelets can also release a large amount of TGF-β, downregulate the NKG2D receptor expression of natural killer (NK) cells, and actively inhibit the function of NK cells (Kopp et al., 2009). Similarly, platelet-stored 5-HT can drive the polarization of macrophages from M1 to the immunosuppressive M2 phenotype and enhance Treg proliferation, synergizing with TGF-β to reinforce immune tolerance (de Las Casas-Engel and Corbí, 2014; Wu et al., 2025). In addition to these soluble factors, platelets can act as carriers of immune checkpoints. For instance, platelets can acquire PD-L1 from tumor cells via a fibronectin–integrin α5β1–GPIbα-dependent mechanism, enabling them to inhibit T cell activity (Hinterleitner et al., 2021). Moreover, platelets form a coating around CTCs, which not only physically blocks the contact of NK cells, but also transfers their MHC-I molecules to the surface of CTCs through membrane exchange, giving them a pseudo-normal phenotype to avoid recognition (Placke et al., 2012).

### 3.4 Tumor-educated platelets

Accumulating evidence indicates that persistent exposure to tumor-derived signals progressively educates circulating platelets through the exchange of molecular cargo, internal RNA processing, and receptor modifications, giving rise to a distinct population known as tumor-educated platelets (TEPs) (In ’t Veld and Wurdinger, 2019; Li Z. et al., 2026). These TEPs display altered transcriptomic and proteomic signatures and acquire capabilities that actively facilitate tumor growth and progression. Current evidence supports that the formation of TEPs may be a combination of multiple mechanisms, including the incorporation of tumor-derived biomolecules (such as proteins, nucleic acids, and extracellular vesicles), specific mRNA splicing events induced by tumor-related stimuli, and the influence of tumor factors on the megakaryocyte production program. Among them, tumor-secreted small extracellular vesicles (sEVs) have been confirmed to be taken up by platelets and carry tumor-related RNA markers into platelets, thereby changing their transcriptome/post-transcriptional regulatory state (Nilsson et al., 2011), and can enhance platelet hyperreactivity and prothrombotic potential through a “non-classical” pathway that is different from the classical ADP/thrombin activation (Dudiki et al., 2023). This provides the biological and methodological basis for cancer liquid biopsy (Best et al., 2015; Ji et al., 2025). For instance, a pan-cancer study showed that TEP RNA-based blood tests enable the detection of 18 cancer types with the potential to identify the tumor site of origin (Veld et al., 2022).

The interaction between platelets and tumor cells activates platelets through both direct contact and soluble mediators or microvesicles, which leads to tumor cell-induced platelet aggregation (TCIPA). In terms of direct contact, podoplanin (PDPN) on the surface of tumor cells or tumor-associated stromal cells can bind to C-type lectin-like receptor 2 (CLEC-2) on platelets, triggering Syk-mediated signaling cascade and promoting aggregation (Suzuki et al., 2022; Sheng et al., 2024). In addition, galectin-3 expressed by tumor cells can be used as the corresponding ligand of platelet GPVI, using the GPVI-FcRγ ITAM-Syk axis to enhance platelet-tumor cell adhesion, aggregation and promote extravasation (Mammadova-Bach et al., 2020). In terms of soluble mediators, a variety of agonists released by the tumor microenvironment can amplify the platelet activation reaction. For example, the tissue factor-related coagulation cascade can lead to thrombin generation, which strongly activates platelets through G protein-coupled receptors such as platelet PARs (Weiser et al., 2025). At the same time, ADP released by platelets forms a positive feedback loop with secondary mediators such as TXA2 to further stabilize the aggregates (Morales-Pacheco et al., 2025).

## 4 The crosstalk between platelet aging and cancer progression

Although the interplay between platelet aging and cancer progression remains largely unexplored, emerging evidence supports a close functional link between them. Currently, most studies treat circulating platelets as a homogeneous population, ignoring their age or senescence status. However, platelets undergo progressive senescence in circulation, characterized by desialylation, PS exposure, and enhanced proinflammatory/procoagulant phenotypes, which may confer distinct functions upon senescent platelets compared to young ones during tumor progression. For instance, desialylation promotes the recognition and clearance of senescent platelets by the hepatic Ashwell-Morell receptor (Grozovsky et al., 2015), a process that activates Kupffer cells and induces systemic immunosuppression (Li et al., 2023), potentially remodeling the liver and systemic immune microenvironments in cancer. PS exposure, which is coupled to the intrinsic apoptosis machinery and to the procoagulant activity of platelets (Schoenwaelder et al., 2009), may enhance interactions between platelets, tumor cells and immune cells (Gay and Felding-Habermann, 2011). A recent single-cell atlas of platelets in aging and cancer provides initial evidence for this heterogeneity-based view by identifying functionally distinct platelet subtypes and showing that an aging-associated Tpm2-high subtype promotes lung metastasis in mice (Li R. et al., 2026). Conversely, cancer and its treatments can alter platelet function and influence aging-like changes. For example, Kawano et al. revealed that tumor-derived, tissue factor-positive extracellular vesicles activate platelets and alter parameters such as platelet count, mean platelet volume, and reticulated platelet percentages (Kawano et al., 2023). Rousseau et al. showed that doxorubicin chemotherapy induces platelet aging-like phenotypes, such as GPIbα shedding (Rousseau et al., 2026). Incorporating platelet senescence into tumor-platelet crosstalk could thus resolve long-standing prognostic discrepancies and inform targeted anti-cancer therapies.

## 5 Conclusion and outlook

Aging-associated platelet hyperreactivity is a risk factor for venous thromboembolism, which is the second leading cause of death among cancer patients (Zöller, 2025). Patients with cancer exhibit enhanced platelet reactivity (Cooke et al., 2013; Pyo et al., 2016), yet thrombocytopenia is also a common complication in cancer patients (Liebman, 2014), likely reflecting a decoupling of platelet count from function in cancer. This review summarizes key aspects of platelet biology and highlights their roles in aging and cancer progression. Beyond traditional hemostatic functions, platelets emerge as dynamic participants in aging and tumor progression. Aged platelets also exhibit increased proinflammatory activity, which may contribute to age-related vascular and inflammatory disorders. Additionally, aged platelets are also hyperactivated, releasing soluble molecules that contribute to the immune evasion of tumor cells. Despite these advances, several important questions remain to be addressed in future research. First, the molecular mechanisms linking platelet aging to altered platelet-tumor crosstalk remain poorly defined. Second, it is unclear whether intervening in aged or hyperactive platelets could promote healthy aging or reduce cancer risk. Third, how the high heterogeneity of platelet count and platelet function independently or synergistically influences aging and cancer progression warrants further investigation.
